# Abnormal Obesity Phenotype Is Associated with Reduced eGFR among Diabetes Mellitus and Hypertensive Patients in a Peri-Urban Community in Ghana

**DOI:** 10.1155/2022/2739772

**Published:** 2022-05-29

**Authors:** Richard K. D. Ephraim, Frederick Ahordzor, Kwame Kumi Asare, Evans Duah, Ibrahim W. Naveh-Fio, Grace Essuman, Justice Afrifa

**Affiliations:** ^1^Department of Medical Laboratory Science, School of Allied Health Sciences, University of Cape Coast, Cape Coast, Ghana; ^2^Department of Biomedical Science, School of Allied Health Sciences, University of Cape Coast, Cape Coast, Ghana

## Abstract

**Background:**

Diabetes mellitus (DM) is a chronic disease characterized by hyperglycemia due to obesity and defects in insulin action. Significant complications of DM include kidney disease due to its association with hypertension and obesity. Thus, the contribution of the various obesity phenotypes to the kidney impairment observed among hypertensive and diabetes mellitus patients is of major concern.

**Aim:**

The study assessed the association between obesity phenotypes and reduced glomerular filtration rate among diabetes mellitus and hypertensive patients.

**Methods:**

Three hundred and ten (310) adult patients diagnosed with type 2 diabetes mellitus, hypertension, or both who attended the Presbyterian Hospital, Dormaa Ahenkro, from October 2016 to March 2017 were recruited for the study. Blood samples were collected to analyze biochemical parameters (fasting blood glucose (FBG), lipid profile, and creatinine). Questionnaires were used to collect sociodemographic information, and anthropometrics were appropriately measured. The estimated glomerular filtration rate (eGFR) was calculated using the CKD-EPI equation, and reduced eGFR was defined as eGFR <90 ml/min/1.73 m^2^.

**Results:**

The prevalence of metabolically healthy nonobese (MHNO), metabolically healthy obese (MHO), metabolically abnormal nonobese (MANO), and metabolically abnormal obese (MAO) phenotypes among the study participants was 30.65%, 4.50%, 52.90%, and 11.94%, respectively. The highest prevalence of reduced eGFR (29/37 (78.38%)) was seen among the MAO group. This was followed by the MANO, MHO, and MHNO with a reduced eGFR prevalence of 62.20%, 57.64%, and 37.89%, respectively. After normalization with MHNO, the reduced eGFR was 1.51, 1.64, and 2.06 times expressed in MHO, MANO, and MAO. For the total samples, when MHNO was maintained as a reference, reduced eGFR was significantly associated with MANO (aOR = 3.07 (95% CI = 1.76–5.35), *P* < 0.001) and MAO (aOR = 5.67 (95% CI = 2.66–17.27), *P* < 0.001) even after adjusting for age, gender, smoking, and alcohol intake. This association was maintained among the female study participants when stratified by gender, and in addition, among the female participants, reduced eGFR was also associated with MHO (aOR = 4.19 (95% CI = 1.06–16.53), *P*=0.041).

**Conclusion:**

There is a high prevalence of abnormal metabolic phenotypes among diabetes mellitus patients, and these were significantly associated with reduced eGFR among our study participants.

## 1. Introduction

Diabetes mellitus consists of a cluster of metabolic diseases marked by elevated glucose levels, which may result from defects in insulin action, insulin secretion, or both. There is a long-standing association between chronic hyperglycemia and long-term dysfunction, damage, and, in most instances, failure of organs such as kidneys, hearts, eyes, and nerves [1]. Propelling the increase in hypertension and diabetes mellitus cases is the heightened prevalence of obesity and overweight in recent years. In combination with infectious diseases, malnutrition and being underweight have been significant problems impacting developing countries [2]. In clinical practice, obesity, classically defined as excess body fat that could lead to a deleterious effect on the body systems, is assessed by the body mass index, which is calculated as the ratio of body weight in kilograms to the body height in square meters [3]. The link between obesity and the upsurge in diabetes mellitus cases is so strong that about 90% of all cases of diabetes mellitus could be attributed to obesity. Again, across the world, obesity and its associated metabolic syndrome have been implicated in the increased level of impaired glucose tolerance shown in approximately 197 million people [4].

Metabolic syndrome is a common metabolic disorder attributed to an increased prevalence of obesity, insulin resistance, and excessive flux of lipids [5]. Furthermore, there is an association between obesity, hypertension, increased mortality, and high prevalence of metabolic disorders as well as organ dysfunction. For instance, obesity, in conjunction with diabetes mellitus and hypertension, has been established as a major precursor to kidney diseases [6]. Lee et al. reported that about one-third of diabetic patients develop diabetic nephropathy and the incidence of this complication keeps increasing, especially in developing countries [7]. However, it is worth noting that not all obese subjects have atypical lipid profile and/or blood pressure levels. There is evidence to show that between 10 and 30% of obese subjects have normal blood pressure or lipid levels, implying there could be a certain number of obese populations that are metabolically healthy [8, 9]. To this end, various researchers have categorized obesity into various phenotypes and assessed its impact on organ function as well as mortality rates [10, 11]. The major categories of the obese phenotypes report include metabolically healthy nonobese (MHNO), defined as having neither MetS nor obesity, metabolically healthy obese (MHO), defined as lacking MetS but being obese, metabolically abnormal nonobese (MANO), defined as having MetS but being nonobese, and finally metabolically abnormal obese (MAO), defined as having MetS and also being obese [12, 13].

Recent studies have demonstrated varying relationships between various obese phenotypes and renal function among different study populations. Among rural people in northeast China, the MHO group was characterized by a higher incidence of mildly decreased glomerular filtration rate [13]. Again, others have shown that, compared to the MHO group, study participants with MAO were associated with a higher risk of developing metabolic diseases and higher mortality [10, 11]. Even though, as stated earlier, diabetic nephropathy remains a hallmark of diabetes mellitus patients, there is a need to assess if the various obesity phenotypes contribute to or compound the renal dysfunction observed among such patients. The study sought to assess the prevalence of the various obesity phenotypes and their association with eGFR among hypertensive and diabetes mellitus patients.

## 2. Methods

### 2.1. Study Design and Sampling

This cross-sectional study was conducted at the diabetes and hypertension clinic of the Dormaa Presbyterian Hospital (DPH), located in the Dormaa Central Municipal Assembly of the Brong Ahafo region. The study involved 310 adult patients diagnosed with either type 2 diabetes mellitus, hypertension, or both who attended the Presbyterian Hospital, Dormaa Ahenkro, from October 2016 to March 2017.

### 2.2. Sample Collection, Preparation, and Biochemical Analysis

Blood specimens were obtained from the antecubital veins of the forearms of the participants after an 8–12-hour overnight fast. Three (3) ml of venous blood samples was collected in a Vacuette Z Serum Sep Clot Activator tube and allowed to clot. The samples were then centrifuged, and the serum was separated and stored till assay. Fasting blood glucose (FBG), total cholesterol (TC), triglycerides (TG), high-density lipoproteins (HDL), and low-density lipoproteins (LDL), urea, and creatinine were analyzed using the Flexor Junior Automated Clinical Chemistry analyzer. The methods adopted for the automated instrument for the determination of the above parameters were according to the reagent manufacturer's instructions (ELITech (SEPPIM S.A.S-Zone Industrielle-61500 SEES FRANCE)).

### 2.3. Anthropometrics

Weight was measured with the subject in light clothing and without shoes, and height was measured with a wall-mounted ruler. The body mass index (BMI) was calculated by dividing weight (kg) by height squared (m^2^). Waist circumference was measured with a plastic anthropometric tape on the bare skin of standing subjects during midrespiration at the bending point at the narrowest indentation midway between the lowest rib and the iliac crest and at the level of the umbilicus to the nearest 0.1 cm.

### 2.4. Blood Pressure

Systolic blood pressure (SBP) and diastolic blood pressure (DBP) were obtained with a standard mercury sphygmomanometer and auscultatory methods. Two blood pressure recordings were obtained from the right arm of each patient in a sitting position, after 30 minutes of rest at 5-minute intervals, and their average value was calculated in accordance with the recommendation of the American Heart Association [14].

### 2.5. Inclusion and Exclusion Criteria

A patient diagnosed with type 2 diabetes mellitus, hypertension, or both who visited the hospital within the said period was enrolled. Patients with hypertension, diabetes mellitus, or both but with evidence of other comorbidities such as kidney diseases and heart failure were excluded from the study. Patients with type 1 diabetes mellitus, those with gestational diabetes, and those who had not observed at least 8 hours of the overnight fast were also excluded.

### 2.6. Ethical Consideration

Ethical clearance was sought from the ethical committee of the Presbyterian Hospital, Dormaa. All participants consented verbally to participate in the study.

### 2.7. Definitions

The diagnosis of MetS was made following the harmonization of the various criteria after the joint organization meeting in 2009 [15]. Five risk factors were elucidated. These are elevated triglyceride ≥1.7 mmol/L, elevated waist circumference ≥90 cm for men and ≥80 cm for women, reduced HDL-C < 1.0 mmol/L in men and <1.3 mmol/L in women; elevated blood pressure taken as systolic ≥130 and/or diastolic ≥85 mmHg, and raised fasting blood glucose ≥5.6 mmol/L. Metabolic syndrome was confirmed with any three of the five risk factors [15]. The Chronic Kidney Disease Epidemiology Collaboration (CKD-EPI) equation was used to estimate the eGFR [16]. Reduced eGFR was defined as eGFR <90 ml/min/1.73 m^2^. Obesity was defined as a BMI of ≥ 30 kg/m^2^ specific for Sub-Saharan Africa [17].

### 2.8. Statistical Analysis

The data were collected and organized in Microsoft Office Excel 2016 (Microsoft Corporation), and the statistical analyses were performed with GraphPad Prism software, version 8.4.3 (GraphPad Software). Results showing the prevalence and distribution of the various obesity phenotypes were expressed as frequencies and percentages. The average levels of the various biochemical parameters stratified into the various obesity phenotypes are expressed as means ± SD. The unpaired *t*-test and one-way ANOVA with multiple comparisons were used to compare mean values of continuous variables for two or more categories. The association of reduced eGFR with obesity phenotype was assessed using logistic regression, and the results were presented as odds ratio and confidence interval. *P* ≤ 0.05 was considered significant.

## 3. Results

The study enrolled 310 diabetes mellitus patients, of which 224 (78.71%) were females. Most of the study participants were predominantly married, 179 (57.74%) had no formal education, 141 (45.48%) were within the age group of 51–70 (55.81%) and 289 (93.23%) were Christians. A significant number, 240 (77.41%), of participants worked in the informal sector, of which the majority (44%) were farmers. Furthermore, about 66.77% of the participants had a family history of diabetes, whilst most of them were nonsmokers (299, 96.45%) and nonalcohol drinkers (93.87). The distribution of the disease type included 39 (12.58%) T2DM patients, 148 (47.74%) hypertensive patients, and 123 (39.68%) T2DM and HPT comorbidity patients (Table 1).

**Figure 1:**
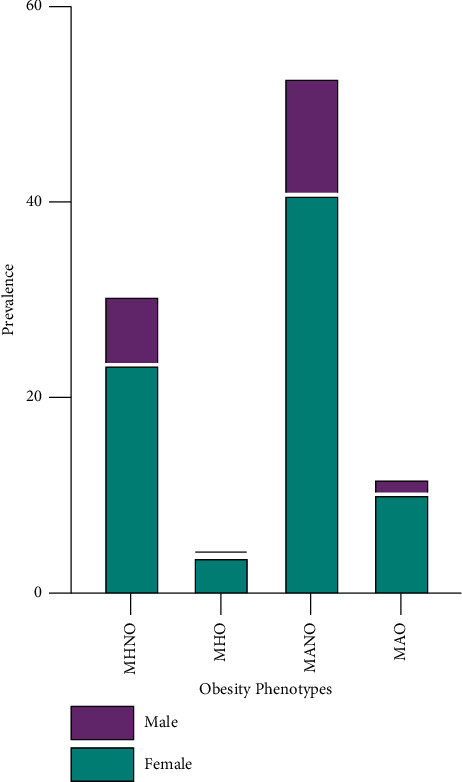
Prevalence of obese phenotypes among the study participants.

The results show that among our study participants, 30.65% (females = 23.55%; males = 7.1%), 4.50% (females = 3.85%, males = 0.65%), 52.90% (more than half) (females = 40.96, males = 11.94%), and 11.94% (females = 10.33%, males = 1.61%) presented with MHNO, MHO, MANO, and MAO, respectively (Figure 1).

Metabolic parameters of the study participants stratified by the different obese phenotypes indicate that the mean BMI (22.06 ± 3.67), WC (87.18 ± 16.89), and TG (1.34 ± 0.38) were significantly reduced (*P* < 0.05) among the MHNO groups compared to the MAO groups. On the contrary, the mean HDL (1.34 ± 0.46^a^) and eGFR (97.48 ± 30.63) were significantly elevated (*P* < 0.05) among the MHNO group compared to the MAO group. Again, eGFR for the MHNO group was significantly lower than that observed for MANO (73.48 ± 22.65) groups. Age stratification showed no significant variation (*P*=0.2139) among the various obese phenotypes. Again, no significant reductions or increases were observed in FBG (*P*=0.2662), TC (*P*=0.5474), and LDL (*P*=0.5030) in the various obese phenotype groups (Table 2).

**Figure 2:**
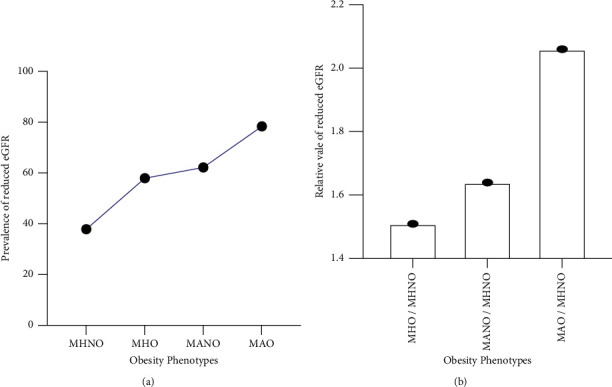
Prevalence (a) and relative expression (b) of reduced eGFR (eGFR < 90) among the various obese phenotypes.

Reduced eGFR was prevalent among the various obese phenotypes at different levels. The highest prevalence, 29/37 (78.38%), was seen among the MAO group. This was followed by the MANO, MHO, and MHNO groups with a reduced eGFR prevalence of 62.20%, 57.94%, and 37.89%, respectively (Figure 2(a)). After normalization with MHNO, the reduced eGFR was 1.51, 1.64, and 2.06 times expressed in MHO, MANO, and MAO, respectively (Figure 2(b)).

eGFR <90 (ml/min/1.73 m^2^) was considered as a reduced kidney function. For the total samples, when MHNO was maintained as reference, reduced eGFR was significantly associated with MANO (aOR = 3.07 (95% CI = 1.76–5.35), *P* < 0.001) and MAO (aOR = 5.67 (95% CI = 2.66–17.27), *P* < 0.001) even after adjusting for age, gender, smoking, and alcohol intake. This association was maintained among the female study participants when stratified by gender, and in addition, among the female participants, reduced eGFR was also associated with MHO (aOR = 4.19 (95% CI = 1.06–16.53), *P*=0.041). However, the association was insignificant among the male participants (Table 3).

## 4. Discussion

Diabetes mellitus and hypertension are two major metabolic diseases that threaten global public health as it approaches epidemic proportions. The global prevalence of these two noncommunicable diseases keeps increasing at a frightening rate. Yearly deaths attributed to cardiovascular diseases for which hypertension and diabetes are significant predisposing factors are estimated at 18 million [18]. Furthermore, numerous studies have established the association between diabetes mellitus and hypertension and kidney diseases with obesity as a cardinal risk factor [19, 20]. However, available evidence shows that not all obese patients may present with an abnormal lipid profile or blood pressure levels [8, 9]. This presupposes that there could be a gap in current information about the effect of the varying obese phenotypes on kidney function. Therefore, the current study assessed the association between the various obesity phenotypes and reduced eGFR among diabetes mellitus and hypertensive patients. The finding showed a high prevalence of abnormal obesity phenotypes (all abnormal phenotypes combined, 69.34%). However, metabolically abnormal nonobese presenting individuals (52.90%) constituted more than half of the study population. This was followed by the MHNO (30.65%), MAO (11.94%), and MHO (4.50%) groups. Reduced eGFR was more prevalent among the MAO (78.38%) and least prevalent among the MHNO (37.89%). Furthermore, there was a significant association of reduced eGFR among the MAO and MANO groups compared to the MHNO group.

Abnormal obesity phenotypes have characterized various apparently healthy populations and thus may even be more pronounced in metabolic diseases such as diabetes mellitus and hypertension. In our study population, more than half of the subjects had one metabolic syndrome but were not obese. Comparatively, in a healthy population, MHO was rather higher among the study participants [13]. This is possible considering that the subjects for this current study include a more significant proportion of hypertensive patients (47.74%) or those suffering from both diabetes mellitus and hypertension (39.68%). Thus, even though they may not be obese, most of them could be presenting with metabolic derangement as evidenced by the MetS.

Kidney dysfunction has been severally associated with metabolic diseases, including diabetes mellitus and hypertension. Lee reported that about one-third of diabetic patients develop diabetic kidney disease and the incidence of this complication keeps increasing, especially in developing countries [7]. In normal populations, the association of obese phenotypes to renal impairment is varied, with reports of the incidence of CKD and reduced renal dysfunction being associated with metabolically healthy obese subjects [21]. Contrary to our findings, there was no significant increased risk of reduced renal function when the MHNO group was compared with the MHO group in the total samples, even though reduced eGFR was about 1.51 times more prevalent in the MHO group. However, when stratified by gender and after adjustment for age, alcohol intake, and smoking, reduced eGFR was significantly associated with MHO among females. This finding supports earlier studies [13] and also gives credence to the assertion that obesity alone, irrespective of other abnormal metabolic activities, is enough to cause the reduction of renal function. This is expressed in various studies in which higher body weight was directly linked with increased urinary oxalate [22], increased excretion of sodium, phosphate, and uric acid [23], and reduced urine pH [24]. It is important to note that unlike the previous studies [13] conducted in the normal population, in this current study, participants are all either diabetics, hypertensives, or both and therefore reduced renal function is expected to be a cardinal hallmark even in the metabolically healthy nonobese group (37.89%).

For the associated risk factors, there seems to be some level of inconclusiveness with regards to their association with renal abnormalities. Some report that being metabolically healthy with obesity is mostly associated with renal abnormalities [25], while others opine that it is the metabolic abnormalities rather than obesity that are associated with reduced renal function [26]. Consequently, this study found that with the metabolically healthy nonobese group as a reference, both MANO and MHNO were significantly associated with a higher occurrence of reduced renal function. In fact, with respect to the MAO group, the occurrence of reduced renal function was twice as much as what was found in the MHNO group and subjects in the MAO group were about 6.78 times more likely to be associated with a reduced eGFR than those in the MHNO group. These findings are in line with the reports of earlier researchers, even in an apparently healthy population. Among rural north Chinese men, a higher incidence of reduced eGFR was associated with subjects who presented with both obesity and metabolic abnormalities [13]. The differing characteristics of the various obese phenotypes and their attendant effects on organ and metabolic functions have been attributed to the possible differences in the metabolite panels. For example, for MAO and MHO, significant differences have been observed in the characteristics and levels of metabolites such as glycerophosphocholine (GPC), L-kynurenine, glycerol 1-phosphate, glycolic acid, and uric acid [27]. This study may be limited by the small sample size, making some groups very small for further analysis. Again, only a single blood test for creatinine was used for the calculation of eGFR, which could introduce some form of bias in the study.

## 5. Conclusion

In conclusion, this study observed a high prevalence of abnormal metabolic phenotypes among diabetes mellitus and hypertensive patients, and these were significantly associated with reduced eGFR among our study participants.

## Figures and Tables

**Table 1:** Prevalence and distribution of the various sociodemographic parameters among the study participants stratified by obesity phenotypes.

Parameters	MHNO	MHO	MANO	MAO	Total
	(*N* = 95)	(*N* = 14)	(*N* = 164)	(*N* = 37)	(*N* = 310)
Age (years)					
≤50	17 (17.89)	5 (35.71)	34 (20.73)	8 (21.62)	64 (20.64)
51–70	53 (55.79)	8 (57.14)	91 (55.49)	21 (56.75)	173 (55.81)
>70	25 (26.32)	1 (7.14)	39 (23.78)	8 (21.62)	73 (23.55)
Gender					
Male	22 (23.16)	2 (14.29)	37 (22.56)	5 (13.51)	66 (21.29)
Female	73 (76.84)	12 (85.71)	127 (77.44)	32 (86.49)	244 (78.71)
Marital status					
Married	55 (57.89)	9 (64.29)	95 (57.93)	20 (54.05)	179 (57.74)
Single	3 (3.16)	0 (0.00)	1 (0.61)	1 (2.70)	5 (1.61)
Divorced	10 (19.53)	3 (21.42)	20 (12.20)	3 (8.11)	36 (11.61)
Widowed	27 (28.42)	2 (14.28)	48 (29.27)	13 (35.14)	90 (29.03)
Educational status					
None	46 (48.42)	2 (14.29)	75 (45.73)	17 (45.95)	140 (45.16)
Primary	40 (42.11)	12 (85.71)	75 (45.73)	19 (51.35)	146 (47.10)
Secondary	2 (2.22)	0 (0.00)	3 (0.61)	0 (0.00)	5 (1.61)
Tertiary	7 (7.37)	0 (0.00)	11 (6.71)	1 (2.70)	19 (6.13)
Occupation					
Formal	6 (6.32)	13 (92.86)	10 (6.10)	1 (2.70)	30 (9.68)
Informal	74 (77.89)	0 (79.41)	122 (74.39)	31 (83.78)	227 (73.23)
Retired	6 (6.32)	0 (0.00)	8 (4.87)	0 (0.00)	14 (4.52)
Unemployed	9 (9.47)	1 (7.14)	24 (14.63)	5 (13.51)	39 (12.58)
Religion					
Christianity	92 (96.84)	12 (94.12)	152 (92.68)	33 (89.19)	289 (93.23)
Muslim	2 (2.11)	2 (5.88)	12 (7.31)	4 (10.81)	20 (6.45)
None	1 (1.05)	0 (0)	0 (0)	0 (1.12)	1 (0.32)
Disease type					
T2DM	14 (14.74)	4 (28.57)	20 (12.20)	1 (2.70)	39 (12.58)
HPT	51 (53.68)	3 (21.43)	74 (45.12)	20 (12.20)	148 (47.74)
T2DM + HPT	30 (31.57)	7 (50.00)	70 (42.68)	16 (9.76)	123 (39.68)
Family history					
Yes	67 (70.72)	12 (85.71)	107 (65.24)	21 (56.76)	207 (66.77)
No	24 (25.26)	2 (14.29)	50 (46.72)	12 (32.43)	88 (28.39)
Unknown	4 (4.21)	0 (0.00)	7 (6.54)	4 (10.81)	15 (4.84)
Smoking					
Yes	1 (1.05)	1 (7.14)	7 (4.26)	2 (5.41)	11 (3.55)
No	94 (98.95)	13 (92.86)	157 (95.73)	35 (94.59)	299 (96.45)
Alcohol					
Yes	4 (4.21)	0 (0.00)	12 (7.32)	3 (8.10)	19 (6.12)
No	91 (95.79)	14 (100.00)	152 (92.68)	34 (91.89)	291 (93.87)

**Table 2:** Metabolic parameters of metabolic healthy nonobese (MHNO), metabolically healthy obese (MHO), metabolic abnormal nonobese (MANO), and metabolic abnormal obese (MAO) subjects.

Total (*N* = 310)	MHNO (*N* = 95)	MHO (*N* = 14)	MANO (*N* = 164)	MAO (*N* = 37)	*P* value
Age (years)	61.76 ± 13.22	54.21 ± 10.67	60.93 ± 12.25	60.41 ± 12.17	0.2139
BMI (kg/m^2^)	22.06 ± 3.67^a^	24.86 ± 4.625^b^	24.31 ± 3.41^c^	34.68 ± 8.50^b^	<0.0001
WC (cm)	87.18 ± 16.89^a^	101.50 ± 15.75^b^	92.51 ± 11.56^bc^	107.1 ± 10.02^c^	<0.0001
SBP (mmHg)	131.9 ± 19.26^a^	137.9 ± 29.40^b^	140.7 ± 17.11^ab^	136.5 ± 18.89^ab^	0.0041
DBP (mmHg)	77.32 ± 10.54^a^	82.14 ± 15.28^ab^	82.14 ± 11.26^b^	84.98 ± 11.41^b^	0.0009
FBG (mmol/L)	7.38 ± 3.53	8.95 ± 4.78	7.63 ± 3.06	6.99 ± 2.86	0.2662
TC (mmol/L)	4.51 ± 1.01	4.48 ± 1.02	4.52 ± 1.09	4.79 ± 1.20	0.5474
TG (mmol/L)	1.34 ± 0.38^a^	1.23 ± 0.20^a^	1.80 ± 0.70^b^	1.85 ± 0.64^b^	<0.0001
HDL-C (mmol/L)	1.34 ± 0.46^a^	1.39 ± 0.42^ac^	1.06 ± 0.38^bc^	1.20 ± 0.36^b^	<0.0001
LDL-C (mmol/L)	2.56 ± 0.93	2.52 ± 0.88	2.64 ± 1.04	2.85 ± 1.10	0.5030
eGFR (ml/min/1.73 m^2^)	97.48 ± 30.63^a^	87.44 ± 46.39^ab^	76.99 ± 24.13^b^	73.48 ± 22.65^b^	<0.0001

Mean values with different superscripts (alphabets) are significantly different.

**Table 3:** Association between reduced eGFR and obese phenotype in different genders.

	Crude model^a^		Adjusted model^b^	
	OR (95% CI)	*P* value	AOR (95% CI)	*P* value
Total				
MHNO	1.00 (reference)		1.00 (reference)	
MHO	2.19 (0.70–6.81)	0.178	3.17 (0.93–10.77)	0.064
MANO	2.69 (1.60–4.54)	**<0.001**	3.07 (1.76–5.35)	**<0.001**
MAO	5.94 (2.45–14.40)	**<0.001**	6.78 (2.66–17.27)	**<0.001**
Female				
MHNO	1.00 (reference)		1.00 (reference)	
MHO	2.71 (0.75–9.81)	0.129	4.19 (1.06–16.53)	**0.041**
MANO	2.65 (1.46–4.78)	**0.001**	3.15 (1.68–5.89)	**<0.001**
MAO	7.32 (2.53–21.14)	**<0.001**	8.96 (2.98–26.99)	**<0.001**
Male				
MHNO	1.00 (reference)		1.00 (reference)	
MANO	3.22 (0.98–10.56)	0.054	2.87 (0.84–9.79)	0.093
MAO	2.27 (0.29–17.58)	0.434	2.82 (0.33–24.29)	0.346

Significance exist at *P* value <0.050. ^a^Crude model: unadjusted. ^b^Adjusted model: adjusted for age, gender, current smoking, and current drinking status.

## Data Availability

Data used for the estimation of means and other calculations will be deposited at the library of the Department of Medical Laboratory Scientists and will be made available upon request.
